# Data assessing genotypic variations in selected traditional rice landraces of Jeypore tract of Odisha, India based on photosynthetic traits

**DOI:** 10.1016/j.dib.2019.104305

**Published:** 2019-08-16

**Authors:** Debabrata Panda, Tanmayee Sahu, Jijnasa Barik, Swati S. Mishra, Bandana Padhan, Sangram K. Lenka

**Affiliations:** a Department of Biodiversity and Conservation of Natural Resources, Central University of Orissa, Koraput, 764 021, Odisha, India; b TERI-Deakin NanoBiotechnology Centre, The Energy and Resources Institute, Gurugram, Haryana, 122 001, India

**Keywords:** Gas exchange, Photosynthesis, Photosystem II activity, Traditional rice, Stomatal conductance

## Abstract

Variations in photosynthetic characteristics and dry matter accumulation were investigated in thirty selected rice (*Oryza sativa* L.) landraces from Jeypore tract of Odisha, India to find the possibility of their use in crop improvement programs. Leaf gas exchange measurements, photosystem (PS) II activity and leaf pigment estimates were conducted at the flowering stage. Significant differences were noticed in the CO_2_ photosynthetic rate (P_N_), stomatal conductance (g_s_), transpiration rate (E), internal CO_2_ concentration (*C*i), water use efficiency (WUE) and carboxylation efficiency (CE) among the landraces. In addition, significant variation was observed in leaf chlorophyll content, PS II activity and dry matter accumulation (DMA). Further, multiple correlations between photosynthetic characteristics and other physiological traits revealed that leaf photosynthesis was not significantly influenced by PS II photochemical activity, leaf area and pigment contents but it was regulated by stomatal conductance, water use efficiency and carboxylation efficiency. Taken together, data presented here shows that some of the landraces had superior photosynthetic traits along with better DMA under prevailing environmental condition and can be used for future crop improvement programs aimed for an increase of leaf photosynthesis in rice.

**Table:** Specifications table

Subject area	Biology
More specific subject area	Plant Physiology
Type of data	Table, figures
How data was acquired	Leaf gas exchange was measured by using an open system photosynthetic gas analyzer (CI-304, CID, USA). Chlorophyll fluorescence was measured by using a portable chlorophyll fluorometer (JUNIOR-PAM, WALZ, Germany). SPAD chlorophyll index was measured by using an SPAD 502 chlorophyll meter (Konica Minolta Sensing, Inc., Osaka, Japan).
Data format	Raw and analyzed data
Experimental factors	Rice landraces were sown directly in earthen pots and were regularly irrigated with tap water and subjected to natural solar radiation. All the measurements were performed three times during flowering stage.
Experimental features	Determination of leaf gas exchange parameters, Different chlorophyll fluorescence parameters, SPAD chlorophyll index, Leaf Pigments, Flag leaf area (LA) and dry matter accumulation (DMA).
Data source location	Experimental garden of Central University of Orissa, Koraput, India (82°44̕ʹ54ʹʹ E to 18°46̕ʹ47ʹʹ N).
Data accessibility	Data is available with this article
Related research article	Mishra et al. [1], Genotypic variability for drought tolerance-related morpho-physiological traits among indigenous rice landraces of Jeypore tract of Odisha, India. Journal of Crop Improvement, 33 (2019) 254–278, 10.1080/15427528.2019.1579138

**Table:** 

**Value of the Data** • First open access visual featured database on variations in photosynthetic characteristics of traditional rice landraces of Jeypore tract of Odisha during flowering stage. • Our data depicts the relationship between leaf photosynthetic parameters and other physiological traits which is highly important to understand the importance of the landraces with respect to quality traits. • The data presented can be a benchmark in future crop improvement programs aimed for improving leaf photosynthesis in rice.

## 1 Data

**Fig. 1:**
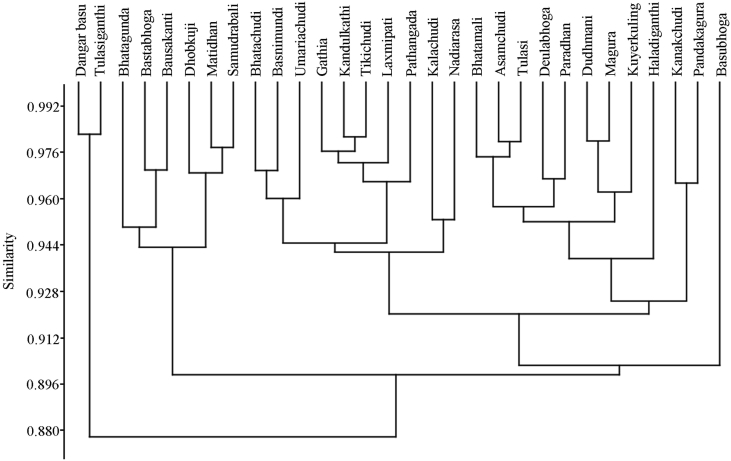
Dendrogram of UPGMA cluster showing the relationship among traditional landraces of Jeypore tract of Odisha based on leaf photosynthetic traits using Bray Curtis similarity index.

**Fig. 2:**
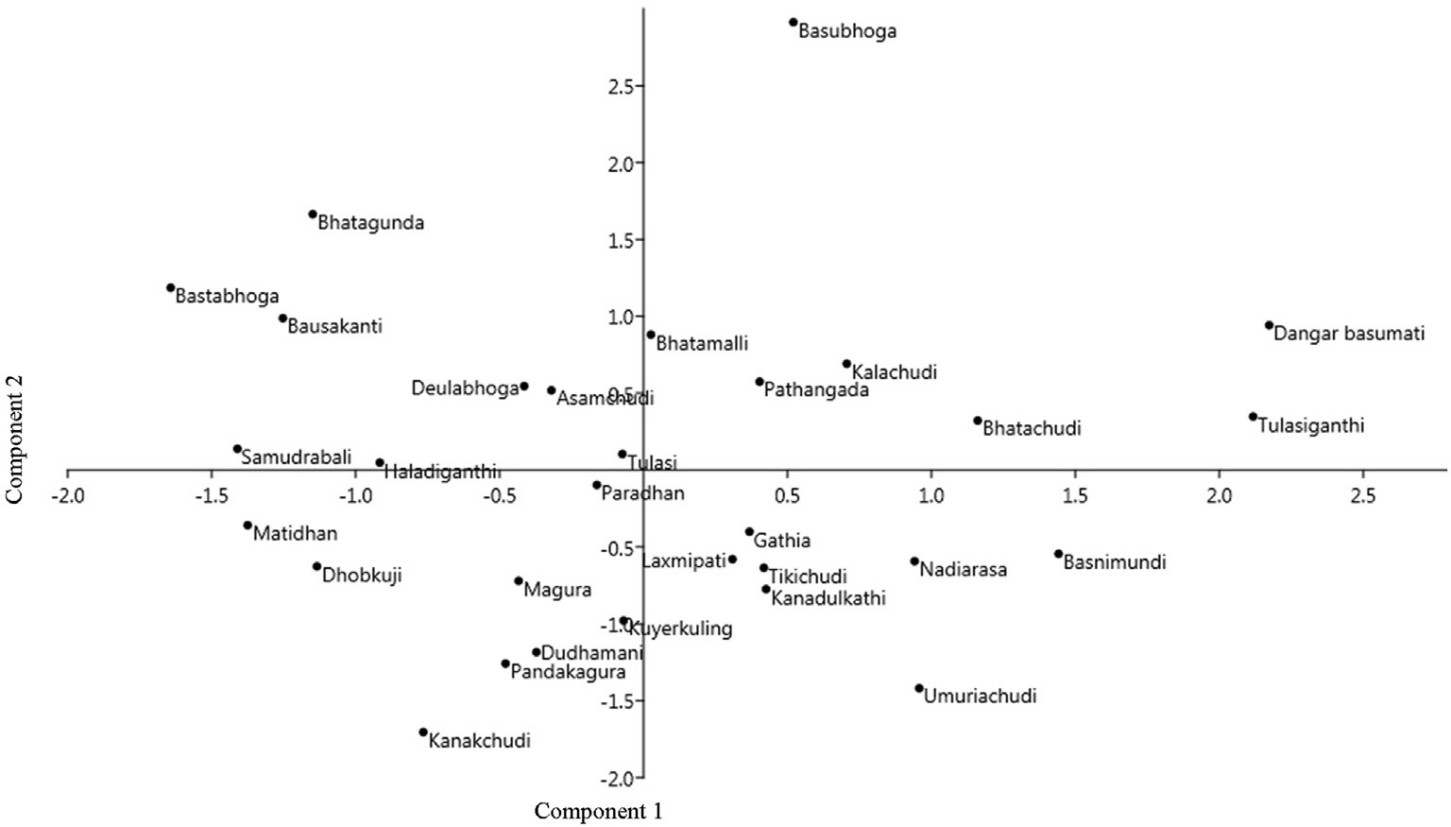
Scatter graph of different traditional rice landraces of Jeypore tract of Odisha measured by using different photosynthetic traits by principal component analysis.

The dataset contains tables and figures on photosynthetic traits of traditional rice landraces of Jeypore tract of Odisha, India. Fig. 1 represents the cluster analysis showing the relationship among traditional landraces of Jeypore tract of Odisha based on leaf photosynthetic traits using Bray Curtis similarity index. Fig. 2 represents scatter graph of different traditional rice landraces and showed that rice landraces ‘Basubhoga’ was most divergent variety followed by ‘Dangarbasumati’ and ‘Tulasiganthi’. List of selected traditional rice landraces with their characteristic feature and habitat type from Jeypore tract of Odisha are described in Table 1. Variations in leaf photosynthetic parameters, leaf pigments and dry matter accumulation in different traditional rice landraces was presented in Table 2. Different chlorophyll fluorescence parameters such as Fo, Fm Fv/Fm, qP and NPQ were presented in Table 3. Significant variation of SPAD index was observed among studied landraces (Table 3).

**Table 1:** List of selected traditional rice landraces with their characteristic feature from Jeypore tract of Odisha.

Landraces	Habitat	Characteristic features
Asamchudi	ML	Popular landrace, maturity duration 125–135 days.^a^,^b^
Basnimundi	LL	Flood resistant, maturity duration 160 days.^b^
Bastabhoga	LL	Aromatic, maturity duration 150 days.^b^
Basubhoga	LL	Aromatic, maturity duration 140 days.^b^
Bausakanti	LL	Flood resistant, maturity duration 155–160 days.^b^
Bhatachudi	ML	Pigmented rice, maturity duration 120–130 days.^b^
Bhatagunda	ML	Popular landrace, maturity duration 120 days.^b^
Bhatamalli	ML	Pigmented rice, maturity duration 120–130 days.
Dangar basumati	UL	Aromatic rice, maturity duration 90–100days.^b^
Deulabhoga	LL	Aromatic rice, maturity duration 140–150 days.^a^
Dhobkuji	LL	Flood resistant, maturity duration 150 days.^b^
Dudhamani	LL	Aromatic rice, maturity duration 140 days.^b^
Gathia	ML	Popular landrace with drought, disease and pest resistant, maturity duration 115–120 days.^b^
Haladiganthi	LL	Pigmented, flood resistant, maturity duration 165 days.^b^
Kalachudi	LL	Popular and pigmented rice.^b^
Kanakchudi	ML	Flood resistant, maturity duration 180 days, good taste and good market price.^a^,^b^
Kanadulkathi	LL	Flood resistant, maturity duration 160 days, pigmented rice.^b^
Kuyerkuling	LL	Popular aromatic rice, maturity duration 140 days, short and oval grain having good market price.^b^
Laxmipati	LL	Profuse tillering, maturity duration 160 days, good taste.^b^
Magura	UL	Popular for drought tolerance.^b^
Matidhan	UL	Popular for drought tolerance and resistant to lodging.^a^
Nadiarasa	LL	Aromatic landrace having maturity duration 140–150 days.^b^
Pandakagura	UL	Aromatic rice, maturity duration 100 days, suitable for multiple cropping, hence a very popular variety.^b^
Paradhan	UL	Pigmented rice, maturity duration 90 days. ≠
Pathangada	LL	Highly preferred popular rice, maturity duration 140–145 days, good yield.^b^
Samudrabali	LL	Aromatic, maturity duration 180 days.^b^
Tikichudi	LL	Aromatic, pigmented rice, maturity duration 110 days.^b^
Tulasi	LL	Aromatic rice, used in socio-cultural and religious ceremonies.^b^
Tulasiganthi	LL	Popular landraces, maturity duration 150–160 days.^b^
Umuriachudi	LL	Most popular landrace, maturity duration 145–150 days.^b^

LL: lowland; ML: medium land; UL: upland.

a Arunachalam et al., [2].

b Mishra et al., [3].

**Table 2:** Variation in leaf photosynthetic characteristics, leaf pigments and dry matter accumulation in traditional rice landraces. Data are the mean of three replications ± standard deviation. Means followed by a common letter in the same column are not significantly different at the 5% level by *Fisher's* least significance difference (LSD) test.

Variety	P_N_	E	g_s_	Ci	WUE	CE	Chl	CAR	LA	DMA
Asamchudi	14.35±0.4^b^	3.00±0.7^c^	66.7±1.4^b^	423.8±1.4^a^	4.79±1.0^b^	0.03±0.001^c^	0.69±0.14^b^	0.098±0.01^a^	3.00±0.28^b,c^	12.2±1.3^d^
Basnimundi	10.80±0.5^d^	2.53±0.4^c^	56.1±2.7^c^	262.8±4.2^d,e^	4.27±1.2^b^	0.04±0.002^b,c^	0.84±0.13^a,b^	0.104±0.05^a^	3.59±0.14^b,c^	11.6±1.0^d^
Bastabhoga	15.60±0.4^b^	2.71±0.4^c^	87.1±4.2^a^	427.1±4.2^a^	5.77±1.1^a,b^	0.04±0.002^b,c^	0.70±0.16^a,b^	0.088±0.06^a^	3.85±0.14^a,b^	12.1±1.5^d^
Basubhoga	16.17±0.4^a,b^	2.07±0.4^c^	74.5±1.4^a,b^	352.1±4.2^b,c^	7.83±1.4^a^	0.05±0.001^a,b^	0.50±0.15^c^	0.072±0.01^a^	1.87±0.14^b,c^	14.0±0.8^c,d^
Bausakanti	17.38±0.3^a^	3.31±0.4^b,c^	92.3±1.4^a^	397.6±4.2^a,b^	5.25±1.5^a,b^	0.04±0.005^b,c^	0.90±0.14^a^	0.118±0.04^a^	1.71±0.08^b,c^	18.4±1.1^a,b^
Bhatachudi	11.58±1.0^c^	2.96±0.4^c^	64.8±2.8^b,c^	287.0±4.2^c,d^	3.92±0.6^b,c^	0.04±0.004^b,c^	0.41±0.14^c^	0.075±0.02^a^	4.52±0.14^a,b^	14.5±1.0^c,d^
Bhatagunda	13.41±1.1^b,c^	2.96±0.4^c^	70.1±2.8^a,b^	415.9±2.8^a^	4.53±0.3^b^	0.03±0.003^c^	0.86±0.12^a,b^	0.112±0.03^a^	4.19±0.01^a,b^	13.1±0.7^c,d^
Bhatamalli	14.22±0.7^b,c^	2.40±0.4^c^	68.0±2.8^b,c^	466.3±2.1^a^	5.94±0.3^a,b^	0.03±0.003^c^	0.62±0.06^b^	0.090±0.01^a^	1.83±0.04^b,c^	16.5±1.5^b,c^
Dangar basumati	10.83±1.1^d^	3.81±0.7^b^	56.0±2.8^c^	340.8±2.8^b,c^	2.84±0.3^c^	0.03±0.001^c^	0.63±0.13^b^	0.081±0.04^a^	3.11±0.01^b^	10.7±0.9^d^
Deulabhoga	18.42±0.6^a^	3.92±0.7^b^	94.4±2.8^a^	370.2±2.8^b,c^	4.70±0.3^b^	0.05±0.005^a,b^	0.35±0.14^b,c^	0.063±0.02^a^	4.05±0.03^a,b^	18.5±0.3^a,b^
Dhobkuji	12.80±1.1^b,c^	3.26±0.7^b,c^	62.6±1.4^b,c^	301.0±1.4^c,d^	3.93±0.3^b,c^	0.04±0.004^b,c^	0.77±0.15^a,b^	0.109±0.03^a^	3.75±0.03^a,b^	14.2±1.1^c,d^
Dudhamani	10.59±0.9^d^	2.87±0.8^b,c^	54.1±1.4^c^	308.7±1.2^c,d^	3.70±1.0^c^	0.03±0.002^c^	0.95±0.13^a^	0.122±0.01^a^	1.34±0.01^b,c^	13.9±1.1^c,d^
Gathia	17.91±1.2^a^	2.33±0.7^c^	99.5±1.4^a^	310.3±1.5^c,d^	7.70±0.9^a^	0.06±0.001^a,b^	0.26±0.14^c^	0.057±0.04^a^	3.56±0.14^a,b^	18.4±1.1^a,b^
Haladiganthi	18.79±1.0^a^	3.27±0.7^b,c^	87.5±1.4^a^	335.0±1.4^b,c^	7.75±1.0^a^	0.06±0.006^a,b^	0.33±0.14^c^	0.062±0.01^a^	5.51±0.14^a^	19.2±1.2^a^
Kalachudi	10.66±0.9^d^	4.21±0.7^a^	78.5±1.4^a,b^	401.6±1.2^a,b^	2.53±1.0^c^	0.03±0.007^c^	0.76±0.15^c^	0.090±0.02^a^	4.15±0.14^a,b^	11.8±1.1^c,d^
Kuyerkuling	12.30±0.4^c,d^	3.15±0.1^b,c^	51.5±0.7^c^	369.3±1.4^b,c^	3.90±1.0^b,c^	0.03±0.002^c^	0.56±0.13^b,c^	0.084±0.01^a^	1.89±0.01^b,c^	16.2±1.0^b,c^
Kanakchudi	10.59±1.1^d^	2.61±0.7^b,c^	41.7±1.2^c,d^	178.6±2.8^d,e^	4.06±1.0^b^	0.06±0.003^a,b^	0.74±0.15^a,b^	0.091±0.02^a^	1.96±0.04^b,c^	12.0±0.5^c,d^
Kanadulkathi	11.07±0.7^c,d^	3.33±0.1^b^	52.5±1.4^c^	297.5±4.2^c,d^	3.33±1.0^a,c^	0.04±0.002^b,c^	0.42±0.13^c^	0.072±0.03^a^	2.81±0.01^b,c^	12.4±0.2^c,d^
Laxmipati	17.51±0.8^a^	3.54±0.4^b^	85.1±1.4^a,b^	359.7±1.4^b,c^	6.95±0.4^a,b^	0.07±0.002^a^	0.51±0.14^b,c^	0.085±0.01^a^	3.00±0.71^a,b^	19.2±1.1^a^
Magura	17.66±1.1^a^	3.57±0.1^b^	62.0±1.4^b,c^	341.0±2.1^b,c^	4.95±0.4^a,b^	0.05±0.005^a,b^	1.03±0.14^a^	0.125±0.02^a^	2.39±0.03^b,c^	19.2±1.4^a^
Matidhan	16.99±0.4^a,b^	3.55 ±0.6^b^	52.0±1.4^c^	315.1±2.8^c,d^	4.79±0.4^b^	0.05±0.006^a,b^	0.95±0.13^a^	0.113±0.04^a^	1.61±0.14^b,c^	20.1±1.2^a^
Nadiarasa	12.59±0.3^c,d^	2.56±0.1^c^	25.0±2.8^e^	386.1±2.8^b,c^	8.07±0.4^a^	0.03±0.002^c^	0.76±0.14^a,b^	0.091±0.01^a^	1.50±0.03^b,c^	11.4±1.4^d^
Pandakagura	14.00±0.5^b,c^	4.26±0.2^a^	67.0±4.2^b^	218.7±1.4^d,e^	3.29±0.4^b,c^	0.06±0.005^a,b^	0.42±0.14^c^	0.069±0.03^a^	2.95±0.03^b,c^	14.6±1.6^c,d^
Paradhan	10.80±0.6^d^	4.80±0.6^a^	72.8±1.4^b^	301.1±1.4^c,d^	2.25±0.4^c^	0.04±0.001^b,c^	0.72±0.14^a,b^	0.096±0.03^a^	2.49±0.07^b,c^	12.3±1.2^c,d^
Pathangada	16.24±0.3^a,b^	3.29±0.5^b,c^	50.9±2.8^c,d^	348.5±5.6^b,c^	4.94±0.4^b^	0.05±0.005^a,b^	0.50±0.08^c^	0.072±0.01^a^	3.42±0.28^b^	14.9±1.1^c,d^
Samudrabali	18.08±1.1^a^	3.97±0.4^b^	86.7±1.4^a,b^	326.2±2.8^b,c^	6.55±0.4^a^	0.06±0.003^a,b^	0.73±0.14^a,b^	0.103±0.04^a^	2.60±0.03^b,c^	18.2±1.2^a,b^
Tikichudi	16.86±1.1^a^	3.55±0.4^b^	40.8±4.2^d^	276.2±0.7^c,d^	4.75±0.4^b^	0.06±0.007^a,b^	0.64±0.14^b^	0.085±0.02^a^	5.15±0.14^a^	13.8±1.0^c,d^
Tulasi	15.30±0.4^a,b^	3.49±0.4^b,c^	67.8±1.4^b,c^	376.9±2.8^b,c^	4.38±0.4^b,c^	0.04±0.001^b,c^	0.75±0.14^a,b^	0.089±0.02^a^	1.41±0.14^b,c^	13.7±1.1^c,d^
Tulasiganthi	16.88±1.1^a^	3.06±0.3^b,c^	71.3±1.4^b,c^	321.5±6.3^c,d^	5.53±0.4^b^	0.05±0.002^a,b^	0.57±0.14^c^	0.070±0.02^a^	1.26±0.14^c^	18.5±0.8^a,b^
Umuriachudi	10.60±0.8^d^	3.82±0.4^b^	53.7±1.4^c^	250.2±4.2^d,e^	2.77±0.8^c^	0.04±0.001^b,c^	0.78±0.14^a,b^	0.091±0.01^a^	2.22±1.7^b,c^	13.2±1.1^c,d^
Mean	14.36	3.24	65.9	338.9	4.67	0.04	0.66	0.91	2.86	14.5
LSD (p < 0.05)	2.50	0.70	14.5	50.5	1.8	0.015	0.28	0.08	1.5	2.2
CV (%)	8.6	9.9	13.4	9.0	8.4	5.1	2.7	3.7	15.3	10.0

P_N_: photosynthetic rate [μmol (CO_2_) m^−2^ s^−1^]; E: transpiration rate [mmol (H_2_O) m^−2^ s^−1^]; g_s_: stomatal conductance [mmol (H_2_O) m^−2^ s^−1^]; Ci: internal CO_2_ concentration (μmol mol^−1^); WUE: water use efficiency (P_N_/E); CE: carboxylation efficiency (P_N_/Ci); Chl: chlorophyll [mg g^−1^ Fm]; CAR: carotenoid [mg g^−1^ Fm]; LA: leaf area (cm^2^); DMA: dry matter accumulation (%).CV: coefficient of variance.

**Table 3:** Variation in leaf chlorophyll fluorescence parameters in traditional rice landraces. Data are the mean of three replications ± standard deviation. Means followed by a common letter in the same column are not significantly different at the 5% level by *Fisher's* least significance difference (LSD) test.

Variety	Fo (rel.)	Fm (rel.)	Fv/Fm (rel.)	qP (rel.)	NPQ (rel.)	SPAD (rel.)
Asamchudi	362.50±14.2^c^	1533.00±29.7^b^	0.76±0.07^a^	0.89±0.03^a^	0.05±0.010^c,d^	35.55±1.1^b^
Basnimundi	421.00±19.2^a,b^	1857.50±76.7^a^	0.77±0.04^a^	0.91±0.05^a^	0.05±0.010^c,d^	39.40±1.2^a^
Bastabhoga	406.50±15.2^b^	1265.00±29.7^c^	0.68±0.05^a^	0.97±0.13^a^	0.05±0.006^c,d^	29.30±1.1^c^
Basubhoga	474.50±24.2^a^	1645.00±15.6^a,b^	0.69±0.04^a^	1.05±0.04^a^	0.06±0.007^c,d^	28.20±1.3^c^
Bausakanti	417.00±12.1^a,b^	1338.00±14.1^c^	0.69±0.03^a^	0.94±0.13^a^	0.06±0.008^c,d^	38.40±1.0^a^
Bhatachudi	482.00±41.2^a^	1795.00±43.8^a^	0.73±0.12^a^	0.97±0.04^a^	0.05±0.002^c,d^	41.00±1.1^a^
Bhatagunda	385.50±44.2^b,c^	1374.00±56.5^b,c^	0.72±0.11^a^	0.90±0.06^a^	0.07±0.005^c,d^	31.65±1.1^b,c^
Bhatamalli	366.00±22.8^c^	1605.00±128.7^a,b^	0.77±0.04^a^	0.93±0.05^a^	0.06±0.004^c,d^	38.50±1.2^a,b^
Dangar basumati	481.00±14.2^a^	1896.00±69.7^a^	0.74±0.11^a^	0.88±0.14^a^	0.05±0.003^c,d^	31.90±1.2^b,c^
Deulabhoga	407.00±12.2^b^	1503.00±29.7^b^	0.73±0.12^a^	0.95±0.13^a^	0.05±0.002^c,d^	32.80±1.3^b,c^
Dhobkuji	345.50±18.2^b,c^	1362.50±59.5^b,c^	0.75±0.03^a^	0.99±0.04^a^	0.06±0.006^b,c^	42.45±1.2^a^
Dudhamani	293.50±17.2^d^	1522.00±39.2^a,b^	0.81±0.14^a^	0.92±0.04^a^	0.04±0.004^c,d^	30.90±1.4^b,c^
Gathia	374.00±14.2^b,c^	1657.50±29.7^a,b^	0.77±0.14^a^	0.85±0.05^a,b^	0.07±0.001^c,d^	36.80±1.2^a,b^
Haladiganthi	296.00±15.2^c,d^	1426.50±29.5^b,c^	0.79±0.06^a^	0.90±0.15^a^	0.07±0.002^c,d^	35.60±1.2^a,b^
Kalachudi	411.00±12.4^a,b^	1727.00±29.7^a,b^	0.76±0.16^a^	0.89±0.07^a^	0.06±0.010^b,c^	38.40±1.0^a^
Kuyerkuling	265.50±32.4^d^	1592.00±29.5^b^	0.83±0.14^a^	0.77±0.09^b^	0.07±0.004^c,d^	38.50±1.1^a^
Kanakchudi	352.00±17.2^b,c^	1422.50±45.6^b,c^	0.75±0.04^a^	0.89±0.10^a^	0.05±0.006^c,d^	37.55±1.2^a^
Kanadulkathi	355.00±18.2^b,c^	1669.50±45.6^a,b^	0.79± 0.12^a^	0.75±0.12^b^	0.07±0.007^c,d^	36.70±1.1^a,b^
Laxmipati	401.50±14.2^a,b^	1636.50±45.4^a,b^	0.75±0.01^a^	0.95±0.04^a^	0.04±0.008^d^	34.80±1.3^a,b^
Magura	310.00±24.2^c,d^	1510.50±15.5^b,c^	0.79±0.12^a^	0.82±0.13^b^	0.07±0.002^c,d^	39.30±1.2^a^
Matidhan	358.00±34.2^c^	1314.00±15.3^b,c^	0.73±0.04^a^	0.90±0.02^a^	0.05±0.005^c,d^	36.10±1.3^a,b^
Nadiarasa	306.00±12.2^c,d^	1787.00±15.6^a^	0.83±0.13^a^	0.80±0.04^b^	0.05±0.006^c,d^	36.80±1.4^a,b^
Pandakagura	362.00±11.4^b,c^	1482.00±16.9^b,c^	0.76±0.12^a^	0.87±0.12^a^	0.07±0.001^c,d^	41.75±1.1^a^
Paradhan	409.50±32.8^a,b^	1545.50±16.9^a,b^	0.74±0.15^a^	0.94±0.13^a^	0.10±0.002^b^	37.70±1.0^a^
Pathangada	445.50±21.4^a,b^	1656.00±16.9^a,b^	0.73±0.06^a^	0.88±0.03^a^	0.04±0.001^d^	40.25±1.1^a^
Samudrabali	394.50±12.8^b^	1303.00±16.9^b,c^	0.70±0.13^a^	0.95±0.04^a^	0.10±0.002^b^	42.30±1.3^a^
Tikichudi	389.00±12.2^b^	1660.50±16.9^a,b^	0.77±0.14^a^	0.87±0.07^a^	0.15±0.002^a^	41.55±1.2^a^
Tulasi	365.00±15.2^b,c^	1577.00±16.9^b^	0.77±0.05^a^	0.88±0.10^a^	0.08±0.001^b,c^	34.30±1.2^b,c^
Tulasiganthi	465.50±14.2^a^	1890.50±15.6^a^	0.77±0.13^a^	0.89±0.12^a^	0.06±0.007^b,c^	32.90±1.2^b,c^
Umuriachudi	338.50±12.8^b,c^	1773.00±16.9^a,b^	0.81±0.05^a^	0.91±0.04^a^	0.08±0.004^b,c^	36.80±1.1^a,b^
**Mean**	**382.35**	**1584.22**	**0.75**	**0.90**	**0.076**	**36.61**
**LSD(p < 0.05)**	**60.2**	**260.0**	**0.21**	**0.18**	**0.02**	**5.5**
**CV (%)**	**6.0**	**15.0**	**7.1**	**3.7**	**5.8**	**10.1**

Fo: minimum fluorescence yield obtained with dark-adapted leaf; Fm: maximum Chl fluorescence yield obtained with dark-adapted leaf; Fv/Fm: maximal photochemical efficiency of PS II; NPQ: non-photochemical quenching; qP: photochemical quenching; CV: coefficient of variance.

Multiple correlation analysis was performed between leaf gas exchanges parameters with other physiological traits (Table 4). The results showed that the rate of photosynthesis (P_N_) was not significantly influenced by leaf pigments (Chl and carotenoid contents) and leaf area. A strong positive correlation between P_N_ with gs, CE and WUE (r = 0.509**, 0.579** and 0.544** respectively, P < 0.01) was observed whereas, leaf P_N_ was negatively correlated with Ci (r = −0.245*, P < 0.05).

**Table 4:** Relationship among leaf photosynthetic parameters and other physiological traits in different traditional rice landraces.

Parameter	P_N_	E	g_s_	Ci	WUE	CE	Fo	Fm	Fv/Fm	qP	NPQ	SPAD	Chl	CAR	LA
E	−0.021^ns^	1													
g_s_	0.509**	0.213*	1												
Ci	−0.245*	−0.207*	−0.297*	1											
WUE	0.575**	−0.76**	0.148 ^ns^	0.320*	1										
CE	0.544**	0.156 ^ns^	0.128 ^ns^	−0.64**	0.170 ^ns^	1									
Fo	0.084 ^ns^	−0.07 ^ns^	0.258*	−0.02 ^ns^	0.172 ^ns^	0.052 ^ns^	1								
Fm	0.348*	−0.12 ^ns^	−0.282*	−0.215*	−0.04 ^ns^	−0.13 ^ns^	0.329*	1							
Fv/Fm	0.308*	0.036 ^ns^	−0.442*	−0.12 ^ns^	−0.216*	−0.13 ^ns^	−0.788 ^ns^	0.314*	1						
qP	0.158^ns^	0.015 ^ns^	0.448*	0.008 ^ns^	0.086 ^ns^	0.099 ^ns^	0.572**	−0.252*	−0.73**	1					
NPQ	0.141 ^ns^	0.450*	−0.03 ^ns^	−0.10 ^ns^	−0.264*	0.166 ^ns^	−0.029 ^ns^	0.034 ^ns^	0.040 ^ns^	−0.058 ^ns^	1				
SPAD	−0.053 ^ns^	0.278*	−0.243*	−0.360*	−0.267*	0.269*	−0.328*	−0.054 ^ns^	0.297*	−0.212*	0.166 ^ns^	1			
Chl	−0.187 ^ns^	0.048 ^ns^	−0.311*	0.080 ^ns^	−0.19 ^ns^	−0.213*	−0.255*	−0.239*	0.081 ^ns^	−0.022 ^ns^	−0.002 ^ns^	0.033 ^ns^	1		
CAR	0.127^ns^	0.201*	0.105 ^ns^	−0.02 ^ns^	−0.03 ^ns^	0.176 ^ns^	−0.005 ^ns^	−0.297*	−0.211*	0.165 ^ns^	0.321*	0.284*	0.12 ^ns^	1	
LA	0.143 ^ns^	0.244*	0.304*	0.101 ^ns^	−0.16 ^ns^	0.047 ^ns^	0.079 ^ns^	−0.082 ^ns^	−0.11 ^ns^	0.152 ^ns^	0.243*	0.182 ^ns^	−0.38*	−0.059 ^ns^	1
DMA	0.564**	0.216*	0.165 ^ns^	0.115 ^ns^	0.070 ^ns^	0.314*	−0.120 ^ns^	−0.277*	−0.04 ^ns^	0.105 ^ns^	−0.005 ^ns^	0.034 ^ns^	0.12 ^ns^	−0.008 ^ns^	−0.100 ^ns^

P_N_: photosynthetic rate; E: transpiration rate; g_s_: stomatal conductance; Ci: internal CO_2_ concentration; WUE: water use efficiency; CE: carboxylation efficiency; Fo: minimum fluorescence yield obtained with dark-adapted leaf; Fm: maximum Chl fluorescence yield obtained with dark-adapted leaf; Fv/Fm: maximal photochemical efficiency of PS II; qP: photosynthetic quenching; NPQ: non photosynthetic quenching; Car: carotenoid; Chl: chlorophyll; DMA: dry matter accumulation. Degree of freedom (df) = 90; *: *p < 0.05*, **: *p < 0.01*, ns: non significant.

## 2 Experimental design, materials, and methods

### 2.1 Plant material and growth condition

The study was conducted by taking thirty selected traditional rice landraces from Jeypore tract of Odisha, India. The detail characteristics of the studied landraces are presented in Table 1. All the landraces were sown directly in earthen pots (30 cm in diameter) containing two kg of farm soil and farmyard manure (3:1) in the campus of Central University of Orissa, Koraput, India (82° 44̕ʹ 54ʹʹ E to 18° 46̕ʹ47ʹʹ N, 880 m above the mean sea level and average rain fall of 1500 mm). After germination, the seedlings were thinned and five plants per pot were maintained. Each pot was supplied with 190 mg single super phosphate (P_2_O_5_) and 50 mg murate of potash (K_2_O). N-fertilizer in the form urea at 1 g per pot was applied trice after 10, 30 and 50 days of sowing. Each landraces was planted in three pots and each pot was treated as separate replications. Plants were regularly irrigated with tap water and subjected to natural solar radiation, with daily maximum photosynthetic photon flux density, air temperature and relative humidity being about 1280±20 μ mol m^−2^ s^−1^, 35.6 ± 2 °C and 65–70%, respectively throughout the experiment. All the measurements were performed three times during flowering stage.

### 2.2 Measurement of leaf gas exchange and chlorophyll fluorescence

The leaf gas exchange parameters were measured between 10 and 12 h on fully matured leaves of each plant using an open system photosynthetic gas analyzer under normal ambient environmental condition. The fully matured 2nd and 3rd leaf from each plant were selected and kept inside the chamber under natural irradiance until stable reading was recorded. The measurements were carried out at 32±2 °C, 60–70% relative humidity, 1014 ± 38 μmol m^−2^ s^−1^ photosynthetic active radiation, 370 μmol CO_2_ m^−2^ s^−1^ and 21% O_2_.

Different chlorophyll fluorescence parameters like minimal fluorescence (Fo), maximal fluorescence (Fm), variable fluorescence (Fv = Fm-Fo) and maximum photochemical efficiency of PS II (Fv/Fm) was measured in 20 min dark-adapted leaves. In light adapted leaves at a PPFD of 400 μmol m^−2^ s^−1^ (for 15 min) steady state fluorescence yield (Fs), maximal fluorescence (Fmʹ) after 0.8 s saturating white light pulse and minimal fluorescence (Foʹ) were measured when actinic light was turned off. Quenching value due to non-photochemical dissipation of absorbed light energy (NPQ) and the coefficient for photochemical quenching (qP) was also calculated [4].

### 2.3 Measurement of SPAD chlorophyll index, flag leaf area and dry matter accumulation

SPAD chlorophyll index was measured on the fully expanded leaf of 5 different plants using an SPAD 502 chlorophyll meter on the intensity of light transmitted 650 nm [5]. The total chlorophyll and carotenoid were measured spectro-photometrically by taking absorbance at 663 nm, 645 nm and 470 nm. The Chl and carotenoid content were calculated using the equation of Arnon [6] and Lichtenthaler and. Wellburn [7].

Flag leaf area (LA) was measured in each plant by taking the length and breadth of leaf and calculated by the using the formula:

Leaf area (cm^2^) = 0.67 x length x width

The dry matter accumulation (DMA) was calculated by taking the fresh and dry weight of two different plants in each replication by the following formula

Dry matter accumulation (%) = (Dry weight / Fresh weight) × 100

### 2.4 Statistical analysis

Differences between various parameters were compared by one way analysis of variance (ANOVA) using CROPSTAT (International Rice Research Institute, Philippines) software. The statistical significance of mean of parameters was determined by performing the Fisher's least significance difference (LSD) test. The cluster analysis was carried out by using Bray-Curtis similarity index using PAST-3 (Palaeontological Statistics) software.
