# Supplementary material for: Data assessing genotypic variations in selected traditional rice landraces of Jeypore tract of Odisha, India based on photosynthetic traits
Source: Data Brief. 2019 Aug 16;25:104305. doi: 10.1016/j.dib.2019.104305 (PMC6728262; doi:10.1016/j.dib.2019.104305)
Supplement: Supplementary file 1 — Multimedia component 1 [file mmc1.docx]

**Table S1**: Raw data of leaf photosynthetic characteristics, chlorophyll fluorescence, leaf pigments and dry matter accumulation in traditional rice landraces. P_N_: photosynthetic rate [µmol (CO_2_) m^-2^ s^-1^]; E: transpiration rate [mmol (H_2_O) m^-2^ s^-1^]; g_s_: stomatal conductance [mmol (H_2_O) m^-2^ s^-1^]; Ci: internal CO_2_ concentration (µmol mol^-1^); WUE: water use efficiency (P_N_/E); CE: carboxylation efficiency (P_N_/Ci); Chl: chlorophyll [mg g^-1^ Fm]; CAR: carotenoid [mg g^-1^ Fm]; LA: leaf area (cm^2^); DMA: dry matter accumulation (%);Fo: minimum fluorescence yield obtained with dark-adapted leaf; Fm: maximum Chl fluorescence yield obtained with dark-adapted leaf; Fv/Fm: maximal photochemical efficiency of PS II; NPQ: non-photochemical quenching; qP: photochemical quenching

| **Variety** | **Replication** | **P_N_** | **E** | **gs** | **Ci** | **WUE** | **CE** | **Fo** | **Fm** | **Fv/Fm** | **qP** | **NPQ** | **SPAD** | **Chl** | **CAR** | **LA** | **DMA** |
| --- | --- | --- | --- | --- | --- | --- | --- | --- | --- | --- | --- | --- | --- | --- | --- | --- | --- |
| **Asamchudi** | 1 | 14.72 | 3.70 | 68.10 | 422.4 | 5.80 | 0.031 | 378.3 | 1558.6 | 0.83 | 0.92 | 0.060 | 35.70 | 0.83 | 0.108 | 3.28 | 13.5 |
|  | 2 | 14.39 | 3.00 | 66.70 | 423.8 | 4.79 | 0.030 | 358.5 | 1540.0 | 0.76 | 0.89 | 0.050 | 36.55 | 0.69 | 0.098 | 3.00 | 12.2 |
|  | 3 | 13.94 | 2.30 | 65.30 | 425.2 | 3.78 | 0.029 | 350.7 | 1500.4 | 0.69 | 0.86 | 0.040 | 34.40 | 0.55 | 0.088 | 2.72 | 10.9 |
| **Basnimundi** | 1 | 11.28 | 2.93 | 58.80 | 258.6 | 5.47 | 0.042 | 436.5 | 1932.7 | 0.81 | 0.96 | 0.060 | 40.60 | 0.97 | 0.154 | 3.73 | 12.6 |
|  | 2 | 10.82 | 2.53 | 56.10 | 262.8 | 4.27 | 0.040 | 427.0 | 1860.5 | 0.77 | 0.91 | 0.050 | 39.40 | 0.84 | 0.104 | 3.59 | 11.6 |
|  | 3 | 10.30 | 2.13 | 53.40 | 267.0 | 3.07 | 0.038 | 399.5 | 1779.3 | 0.73 | 0.86 | 0.040 | 38.20 | 0.71 | 0.054 | 3.45 | 10.6 |
| **Bastabhoga** | 1 | 16.00 | 3.11 | 91.30 | 422.9 | 6.87 | 0.042 | 421.7 | 1296.9 | 0.73 | 1.10 | 0.056 | 30.40 | 0.86 | 0.148 | 3.99 | 13.6 |
|  | 2 | 15.60 | 2.71 | 87.10 | 427.1 | 5.77 | 0.040 | 406.5 | 1259.9 | 0.68 | 0.97 | 0.050 | 29.30 | 0.70 | 0.088 | 3.85 | 12.1 |
|  | 3 | 15.20 | 2.31 | 82.90 | 431.3 | 4.67 | 0.038 | 391.3 | 1238.2 | 0.63 | 0.84 | 0.044 | 28.20 | 0.54 | 0.028 | 3.71 | 10.6 |
| **Basubhoga** | 1 | 16.59 | 2.37 | 75.90 | 347.9 | 9.23 | 0.051 | 496.5 | 1660.6 | 0.73 | 1.09 | 0.067 | 29.50 | 0.65 | 0.082 | 2.01 | 14.8 |
|  | 2 | 16.15 | 2.27 | 74.50 | 352.1 | 7.83 | 0.050 | 478.5 | 1645.0 | 0.69 | 1.05 | 0.060 | 28.20 | 0.50 | 0.072 | 1.87 | 14.0 |
|  | 3 | 15.76 | 1.57 | 73.10 | 356.3 | 6.43 | 0.049 | 448.5 | 1629.4 | 0.65 | 1.01 | 0.053 | 26.90 | 0.35 | 0.062 | 1.74 | 13.2 |
| **Bausakanti** | 1 | 17.69 | 3.71 | 93.78 | 393.4 | 6.75 | 0.045 | 427.2 | 1352.1 | 0.72 | 1.07 | 0.068 | 39.40 | 1.04 | 0.158 | 1.79 | 19.5 |
|  | 2 | 17.37 | 3.31 | 92.30 | 397.6 | 5.25 | 0.040 | 420.2 | 1338.0 | 0.69 | 0.94 | 0.060 | 38.40 | 0.90 | 0.118 | 1.71 | 18.4 |
|  | 3 | 17.08 | 2.91 | 90.90 | 401.8 | 3.75 | 0.035 | 403.6 | 1323.9 | 0.66 | 0.81 | 0.052 | 37.40 | 0.76 | 0.078 | 1.63 | 17.3 |
| **Bhatachudi** | 1 | 12.53 | 3.36 | 67.60 | 282.8 | 4.52 | 0.044 | 520.5 | 1838.8 | 0.85 | 1.01 | 0.052 | 42.10 | 0.55 | 0.095 | 4.66 | 15.5 |
|  | 2 | 11.58 | 2.96 | 64.80 | 287.0 | 3.92 | 0.040 | 487.0 | 1795.0 | 0.73 | 0.97 | 0.050 | 41.00 | 0.41 | 0.075 | 4.52 | 14.5 |
|  | 3 | 10.63 | 2.56 | 62.00 | 291.2 | 3.32 | 0.036 | 438.5 | 1751.2 | 0.61 | 0.93 | 0.048 | 39.90 | 0.27 | 0.055 | 4.38 | 13.5 |
| **Bhatagunda** | 1 | 14.50 | 3.38 | 72.90 | 413.1 | 4.83 | 0.033 | 427.0 | 1430.5 | 0.83 | 0.96 | 0.075 | 32.78 | 0.98 | 0.142 | 4.20 | 13.8 |
|  | 2 | 13.42 | 2.96 | 70.10 | 415.9 | 4.53 | 0.030 | 390.5 | 1374.0 | 0.72 | 0.90 | 0.070 | 31.65 | 0.86 | 0.112 | 4.19 | 13.1 |
|  | 3 | 12.31 | 2.54 | 67.30 | 418.7 | 4.23 | 0.027 | 339.0 | 1317.5 | 0.61 | 0.84 | 0.065 | 30.52 | 0.74 | 0.082 | 4.18 | 12.4 |
| **Bhatamalli** | 1 | 14.92 | 2.80 | 70.80 | 464.2 | 6.24 | 0.033 | 391.2 | 1733.7 | 0.81 | 0.98 | 0.064 | 39.70 | 0.68 | 0.100 | 1.87 | 18.0 |
|  | 2 | 14.20 | 2.40 | 68.00 | 466.3 | 5.94 | 0.030 | 360.0 | 1605.0 | 0.77 | 0.93 | 0.060 | 38.50 | 0.62 | 0.090 | 1.83 | 16.5 |
|  | 3 | 13.54 | 2.00 | 65.20 | 468.4 | 5.64 | 0.027 | 346.8 | 1476.3 | 0.73 | 0.88 | 0.056 | 37.30 | 0.56 | 0.080 | 1.79 | 15.0 |
| **Dangar basumati** | 1 | 11.91 | 4.49 | 58.80 | 338.0 | 3.14 | 0.031 | 495.2 | 1963.6 | 0.85 | 1.02 | 0.053 | 33.12 | 0.76 | 0.121 | 3.12 | 11.6 |
|  | 2 | 10.82 | 3.81 | 56.00 | 340.8 | 2.84 | 0.030 | 481.0 | 1900.0 | 0.74 | 0.88 | 0.050 | 31.90 | 0.63 | 0.081 | 3.11 | 10.7 |
|  | 3 | 9.76 | 3.13 | 53.20 | 343.6 | 2.54 | 0.029 | 466.8 | 1824.4 | 0.63 | 0.74 | 0.047 | 30.68 | 0.50 | 0.041 | 3.10 | 9.8 |
| **Deulabhoga** | 1 | 19.03 | 4.62 | 97.20 | 367.4 | 5.00 | 0.055 | 419.2 | 1532.7 | 0.85 | 1.08 | 0.052 | 34.10 | 0.49 | 0.083 | 4.07 | 18.8 |
|  | 2 | 18.43 | 3.92 | 94.40 | 370.2 | 4.70 | 0.050 | 407.0 | 1503.0 | 0.73 | 0.95 | 0.050 | 32.80 | 0.35 | 0.063 | 4.05 | 18.5 |
|  | 3 | 17.80 | 3.22 | 91.60 | 373.0 | 4.40 | 0.045 | 394.8 | 1473.3 | 0.61 | 0.82 | 0.048 | 31.50 | 0.21 | 0.043 | 4.02 | 18.2 |
| **Dhobkuji** | 1 | 13.92 | 3.96 | 64.00 | 299.6 | 4.23 | 0.044 | 363.7 | 1422.0 | 0.78 | 1.03 | 0.066 | 43.63 | 0.92 | 0.139 | 3.78 | 15.3 |
|  | 2 | 12.78 | 3.26 | 62.60 | 301.0 | 3.93 | 0.040 | 345.5 | 1362.5 | 0.75 | 0.99 | 0.060 | 42.45 | 0.77 | 0.109 | 3.75 | 14.2 |
|  | 3 | 11.70 | 2.56 | 61.20 | 302.4 | 3.63 | 0.036 | 327.3 | 1303.0 | 0.72 | 0.95 | 0.054 | 41.27 | 0.62 | 0.079 | 3.72 | 13.1 |
| **Dudhamani** | 1 | 11.48 | 3.67 | 55.50 | 307.5 | 4.68 | 0.032 | 310.7 | 1561.2 | 0.95 | 0.96 | 0.044 | 32.30 | 1.08 | 0.132 | 1.35 | 15.1 |
|  | 2 | 10.59 | 2.87 | 54.10 | 308.7 | 3.70 | 0.030 | 293.5 | 1522.0 | 0.81 | 0.92 | 0.040 | 30.90 | 0.95 | 0.122 | 1.34 | 13.6 |
|  | 3 | 9.70 | 2.07 | 52.70 | 309.9 | 2.72 | 0.028 | 276.3 | 1482.8 | 0.67 | 0.88 | 0.036 | 29.50 | 0.82 | 0.112 | 1.33 | 12.9 |
| **Gathia** | 1 | 19.10 | 3.03 | 100.90 | 308.8 | 8.60 | 0.061 | 388.2 | 1687.2 | 0.91 | 0.90 | 0.071 | 38.00 | 0.40 | 0.017 | 3.70 | 19.5 |
|  | 2 | 17.93 | 2.33 | 99.50 | 310.3 | 7.70 | 0.060 | 374.0 | 1657.5 | 0.77 | 0.85 | 0.070 | 36.80 | 0.26 | 0.057 | 3.56 | 18.4 |
|  | 3 | 16.70 | 1.63 | 98.10 | 311.8 | 6.80 | 0.059 | 359.8 | 1627.8 | 0.63 | 0.80 | 0.069 | 35.60 | 0.12 | 0.097 | 3.42 | 17.3 |
| **Haladiganthi** | 1 | 19.77 | 3.97 | 88.90 | 333.6 | 8.75 | 0.066 | 311.2 | 1456.0 | 0.85 | 1.05 | 0.072 | 36.80 | 0.47 | 0.072 | 5.65 | 20.4 |
|  | 2 | 18.80 | 3.27 | 87.50 | 335.0 | 7.75 | 0.060 | 296.0 | 1426.5 | 0.79 | 0.90 | 0.070 | 35.60 | 0.33 | 0.062 | 5.51 | 19.2 |
|  | 3 | 17.80 | 2.57 | 86.10 | 336.4 | 6.75 | 0.054 | 280.8 | 1397.0 | 0.73 | 0.75 | 0.068 | 34.40 | 0.19 | 0.052 | 5.37 | 18.0 |
| **Kalachudi** | 1 | 11.57 | 4.91 | 79.90 | 400.4 | 3.55 | 0.037 | 420.9 | 1756.7 | 0.92 | 0.96 | 0.070 | 39.43 | 0.91 | 0.110 | 4.29 | 12.9 |
|  | 2 | 10.64 | 4.21 | 78.50 | 401.6 | 2.53 | 0.030 | 415.0 | 1727.0 | 0.76 | 0.89 | 0.060 | 38.40 | 0.76 | 0.090 | 4.15 | 11.8 |
|  | 3 | 9.77 | 3.51 | 77.10 | 402.8 | 1.51 | 0.023 | 397.1 | 1697.3 | 0.60 | 0.82 | 0.050 | 37.37 | 0.61 | 0.070 | 4.01 | 10.7 |
| **Kuyerkuling** | 1 | 12.71 | 3.25 | 52.20 | 367.9 | 4.90 | 0.032 | 297.9 | 1619.3 | 0.97 | 0.86 | 0.074 | 39.50 | 0.69 | 0.093 | 1.90 | 17.2 |
|  | 2 | 12.30 | 3.15 | 51.50 | 369.3 | 3.90 | 0.030 | 265.5 | 1596.0 | 0.83 | 0.77 | 0.070 | 38.50 | 0.56 | 0.084 | 1.89 | 16.2 |
|  | 3 | 11.89 | 3.05 | 50.80 | 370.7 | 2.90 | 0.028 | 233.1 | 1560.7 | 0.69 | 0.68 | 0.066 | 37.50 | 0.43 | 0.075 | 1.88 | 15.2 |
| **Kanakchudi** | 1 | 11.70 | 3.30 | 42.90 | 175.8 | 5.06 | 0.063 | 366.9 | 1468.1 | 0.79 | 0.99 | 0.056 | 38.75 | 0.89 | 0.111 | 2.00 | 12.5 |
|  | 2 | 10.60 | 2.61 | 41.70 | 178.6 | 4.06 | 0.060 | 356.0 | 1422.5 | 0.75 | 0.89 | 0.050 | 37.55 | 0.74 | 0.091 | 1.96 | 12.0 |
|  | 3 | 9.47 | 1.92 | 40.50 | 181.4 | 3.06 | 0.057 | 333.1 | 1376.9 | 0.71 | 0.79 | 0.044 | 36.35 | 0.59 | 0.071 | 1.92 | 11.5 |
| **Kanadulkathi** | 1 | 11.75 | 3.43 | 53.90 | 293.3 | 4.35 | 0.042 | 373.2 | 1715.1 | 0.91 | 0.87 | 0.077 | 37.80 | 0.55 | 0.102 | 2.82 | 12.6 |
|  | 2 | 11.08 | 3.33 | 52.50 | 297.5 | 3.33 | 0.040 | 355.0 | 1669.5 | 0.79 | 0.75 | 0.070 | 36.70 | 0.42 | 0.072 | 2.81 | 12.4 |
|  | 3 | 10.38 | 3.23 | 51.10 | 301.7 | 2.31 | 0.038 | 336.8 | 1623.9 | 0.67 | 0.63 | 0.063 | 35.60 | 0.29 | 0.042 | 2.80 | 12.2 |
| **Laxmipati** | 1 | 18.28 | 3.94 | 86.53 | 358.3 | 7.35 | 0.072 | 415.7 | 1681.4 | 0.76 | 0.99 | 0.048 | 36.10 | 0.65 | 0.095 | 3.71 | 20.3 |
|  | 2 | 17.53 | 3.54 | 85.10 | 359.7 | 6.95 | 0.070 | 401.5 | 1637.5 | 0.75 | 0.95 | 0.040 | 34.80 | 0.51 | 0.085 | 3.00 | 19.2 |
|  | 3 | 16.72 | 3.14 | 83.79 | 361.1 | 6.55 | 0.068 | 387.3 | 1590.6 | 0.74 | 0.91 | 0.032 | 33.50 | 0.37 | 0.075 | 2.29 | 18.1 |
| **Magura** | 1 | 18.77 | 3.67 | 63.40 | 338.9 | 5.35 | 0.055 | 330.6 | 1526.0 | 0.91 | 0.95 | 0.072 | 40.50 | 1.17 | 0.145 | 2.42 | 20.6 |
|  | 2 | 17.63 | 3.57 | 62.00 | 341.0 | 4.95 | 0.050 | 316.0 | 1510.5 | 0.79 | 0.82 | 0.070 | 39.30 | 1.03 | 0.125 | 2.39 | 19.2 |
|  | 3 | 16.58 | 3.47 | 60.60 | 343.1 | 4.55 | 0.045 | 283.4 | 1495.0 | 0.67 | 0.69 | 0.068 | 38.10 | 0.89 | 0.105 | 2.36 | 17.8 |
| **Matidhan** | 1 | 17.40 | 4.15 | 53.40 | 312.3 | 5.19 | 0.056 | 392.2 | 1329.3 | 0.77 | 0.92 | 0.055 | 37.40 | 1.08 | 0.153 | 1.75 | 21.3 |
|  | 2 | 16.97 | 3.55 | 52.00 | 315.1 | 4.79 | 0.050 | 358.0 | 1314.0 | 0.73 | 0.90 | 0.050 | 36.10 | 0.95 | 0.113 | 1.61 | 20.1 |
|  | 3 | 16.60 | 2.95 | 50.60 | 317.9 | 4.39 | 0.044 | 323.8 | 1298.7 | 0.69 | 0.88 | 0.045 | 34.80 | 0.82 | 0.073 | 1.47 | 18.9 |
| **Nadiarasa** | 1 | 12.87 | 2.66 | 27.80 | 383.3 | 8.47 | 0.032 | 315.7 | 1802.6 | 0.96 | 0.84 | 0.056 | 38.20 | 0.90 | 0.102 | 1.53 | 12.8 |
|  | 2 | 12.60 | 2.56 | 25.00 | 386.1 | 8.07 | 0.030 | 310.0 | 1787.0 | 0.83 | 0.80 | 0.050 | 36.80 | 0.76 | 0.091 | 1.50 | 11.4 |
|  | 3 | 12.30 | 2.46 | 22.80 | 388.9 | 7.67 | 0.028 | 292.3 | 1771.4 | 0.70 | 0.76 | 0.044 | 35.40 | 0.62 | 0.080 | 1.47 | 10.0 |
| **Pandakagura** | 1 | 14.50 | 4.46 | 71.20 | 217.3 | 3.69 | 0.065 | 373.4 | 1498.9 | 0.88 | 0.99 | 0.071 | 42.85 | 0.56 | 0.099 | 2.98 | 16.2 |
|  | 2 | 14.00 | 4.26 | 67.00 | 218.7 | 3.29 | 0.060 | 362.0 | 1482.0 | 0.76 | 0.87 | 0.070 | 41.75 | 0.42 | 0.069 | 2.95 | 14.6 |
|  | 3 | 13.50 | 4.06 | 62.80 | 220.1 | 2.89 | 0.055 | 350.6 | 1465.1 | 0.64 | 0.75 | 0.069 | 40.65 | 0.28 | 0.039 | 2.92 | 13.0 |
| **Paradhan** | 1 | 11.41 | 5.40 | 74.20 | 299.7 | 2.65 | 0.041 | 442.3 | 1562.4 | 0.89 | 1.07 | 0.102 | 38.74 | 0.86 | 0.126 | 2.56 | 13.5 |
|  | 2 | 10.82 | 4.80 | 72.80 | 301.1 | 2.25 | 0.040 | 409.5 | 1545.5 | 0.74 | 0.94 | 0.100 | 37.70 | 0.72 | 0.096 | 2.49 | 12.3 |
|  | 3 | 10.17 | 4.20 | 71.40 | 302.5 | 1.85 | 0.039 | 376.7 | 1528.6 | 0.59 | 0.81 | 0.098 | 36.66 | 0.58 | 0.066 | 2.42 | 11.1 |
| **Pathangada** | 1 | 16.55 | 3.79 | 53.70 | 342.9 | 5.34 | 0.055 | 464.0 | 1672.9 | 0.79 | 0.91 | 0.041 | 41.35 | 0.58 | 0.730 | 3.70 | 16.0 |
|  | 2 | 16.26 | 3.29 | 50.90 | 348.5 | 4.94 | 0.050 | 450.5 | 1656.0 | 0.73 | 0.88 | 0.040 | 40.25 | 0.50 | 0.720 | 3.42 | 14.9 |
|  | 3 | 15.91 | 2.79 | 48.10 | 354.1 | 4.54 | 0.045 | 422.0 | 1639.1 | 0.67 | 0.85 | 0.039 | 39.15 | 0.42 | 0.710 | 3.14 | 13.8 |
| **Samudrabali** | 1 | 19.19 | 4.37 | 88.10 | 323.4 | 6.95 | 0.063 | 407.3 | 1315.3 | 0.83 | 0.99 | 0.102 | 43.60 | 0.87 | 0.143 | 2.63 | 19.4 |
|  | 2 | 18.06 | 3.97 | 86.70 | 326.2 | 6.55 | 0.060 | 394.5 | 1310.0 | 0.70 | 0.95 | 0.100 | 42.30 | 0.73 | 0.103 | 2.60 | 18.2 |
|  | 3 | 16.99 | 3.57 | 85.30 | 329.0 | 6.15 | 0.057 | 381.7 | 1283.7 | 0.57 | 0.91 | 0.098 | 41.00 | 0.59 | 0.063 | 2.57 | 17.0 |
| **Tikichudi** | 1 | 17.97 | 3.95 | 45.00 | 275.5 | 5.15 | 0.067 | 401.2 | 1677.4 | 0.91 | 0.94 | 0.152 | 42.75 | 0.78 | 0.105 | 5.29 | 14.8 |
|  | 2 | 16.87 | 3.55 | 40.80 | 276.2 | 4.75 | 0.060 | 389.0 | 1660.5 | 0.77 | 0.87 | 0.150 | 41.55 | 0.64 | 0.085 | 5.15 | 13.8 |
|  | 3 | 15.74 | 3.15 | 36.60 | 276.9 | 4.35 | 0.053 | 376.8 | 1643.6 | 0.63 | 0.80 | 0.148 | 40.35 | 0.50 | 0.065 | 5.01 | 12.8 |
| **Tulasi** | 1 | 15.72 | 3.89 | 69.20 | 374.1 | 4.78 | 0.041 | 380.2 | 1593.9 | 0.82 | 0.98 | 0.081 | 35.50 | 0.89 | 0.110 | 1.56 | 14.8 |
|  | 2 | 15.29 | 3.49 | 67.80 | 376.9 | 4.38 | 0.040 | 365.0 | 1577.0 | 0.77 | 0.88 | 0.080 | 34.30 | 0.75 | 0.089 | 1.41 | 13.7 |
|  | 3 | 14.89 | 3.09 | 66.40 | 379.7 | 3.98 | 0.039 | 349.8 | 1560.1 | 0.72 | 0.78 | 0.079 | 33.10 | 0.61 | 0.068 | 1.28 | 12.6 |
| **Tulasiganthi** | 1 | 18.01 | 3.36 | 72.70 | 315.2 | 5.93 | 0.052 | 476.0 | 1906.1 | 0.90 | 1.01 | 0.067 | 34.10 | 0.71 | 0.090 | 1.40 | 19.3 |
|  | 2 | 16.86 | 3.06 | 71.30 | 321.5 | 5.53 | 0.050 | 470.0 | 1890.5 | 0.77 | 0.89 | 0.060 | 32.90 | 0.57 | 0.070 | 1.26 | 18.5 |
|  | 3 | 15.77 | 2.76 | 69.90 | 327.8 | 5.13 | 0.048 | 449.0 | 1874.9 | 0.64 | 0.77 | 0.053 | 31.70 | 0.43 | 0.050 | 1.12 | 17.7 |
| **Umuriachudi** | 1 | 11.40 | 4.22 | 55.10 | 246.0 | 3.57 | 0.041 | 352.5 | 1785.3 | 0.86 | 0.95 | 0.084 | 37.90 | 0.92 | 0.081 | 3.92 | 14.3 |
|  | 2 | 10.60 | 3.82 | 53.70 | 250.2 | 2.77 | 0.040 | 335.5 | 1780.0 | 0.81 | 0.91 | 0.080 | 36.80 | 0.78 | 0.091 | 2.22 | 13.2 |
|  | 3 | 9.80 | 3.42 | 52.30 | 254.4 | 1.97 | 0.039 | 327.5 | 1753.7 | 0.76 | 0.87 | 0.076 | 35.70 | 0.64 | 0.101 | 0.52 | 12.1 |
